# Light signalling shapes plant–plant interactions in dense canopies

**DOI:** 10.1111/pce.13912

**Published:** 2020-10-22

**Authors:** Martina Huber, Nicole M. Nieuwendijk, Chrysoula K. Pantazopoulou, Ronald Pierik

**Affiliations:** ^1^ Plant Ecophysiology, Dept. Biology Utrecht University Utrecht The Netherlands

**Keywords:** canopy, competition, light, photoreceptor, plant–plant interaction

## Abstract

Plants growing at high densities interact via a multitude of pathways. Here, we provide an overview of mechanisms and functional consequences of plant architectural responses initiated by light cues that occur in dense vegetation. We will review the current state of knowledge about shade avoidance, as well as its possible applications. On an individual level, plants perceive neighbour‐associated changes in light quality and quantity mainly with phytochromes for red and far‐red light and cryptochromes and phototropins for blue light. Downstream of these photoreceptors, elaborate signalling and integration takes place with the *PHYTOCHROME INTERACTING FACTORS*, several hormones and other regulators. This signalling leads to the shade avoidance responses, consisting of hyponasty, stem and petiole elongation, apical dominance and life cycle adjustments. Architectural changes of the individual plant have consequences for the plant community, affecting canopy structure, species composition and population fitness. In this context, we highlight the ecological, evolutionary and agricultural importance of shade avoidance.

## Abstract 2

Causes and consequences of plant–plant signalling through light cues: from genes to canopies.

## 1 INTRODUCTION

Plants growing at high densities compete for light, as well as other primary resources such as water and nutrients. In such a crowded environment, shade‐intolerant plants are able to adjust their development and physiology to optimize resource acquisition in order to escape from these unfavourable conditions. But how does a plant even detect that there are competing neighbours around? Several environmental cues provide information about the presence of competitors: volatile organic compounds that carry information about neighbouring plants through the air (Pierik, Visser, De Kroon, & Voesenek, 2003), below ground root exudates and volatile organic compounds (Guo et al., 2018; Worthington & Reberg‐Horton, 2013) and mechanical interaction via physical touching of neighbour leaves (Wit et al., 2012), all provide information about proximate vegetation. However, the dominant cues for neighbour detection at high planting density are associated with light quality and quantity (Ballaré, 1999; Ballaré & Pierik, 2017; Casal, 2012; Pierik & Wit, 2014; Roig‐Villanova & Martínez‐García, 2016). Clearly, the presence, intensity and reliability of neighbour cues depends on their proximity: At high planting densities, neighbours are very nearby and cues, be it chemical or visual, will be both strong and reliable. At low densities, or very early stages of stand development, most cues will not be sufficiently strong to elicit major responses in receiving plants. Nevertheless, depending on the specific light cues, a plant can detect whether it is truly shaded, for example, by an overhead canopy (foliage shade) or surrounded by neighbours of similar height (changes in light quality) (Wit et al., 2016).

In this review, we will focus primarily on the changes in light quality that serve as early cues for impeding competition and cause responses in neighbours. These trigger a suite of responses that change plant development and architecture, collectively referred to as the shade‐avoidance syndrome (SAS), sometimes even before actual shading occurs (Ballaré, 1999; Ballaré, Scopel, & Sánchez, 1990; Pierik & Wit, 2014; Schmitt, Dudley, & Pigliucci, 1999). In this review, we treat the term ‘canopy’ in its most general sense (Table 1), for which we will discuss changes in traits related to canopy architecture. Primarily, we will be focussing on herbaceous plants; responses of perennial, woody plants are mostly beyond the scope of this review.

**TABLE 1:** Definitions and abbreviations

Adaptation	Adaptation refers to heritable, genotypic traits – in contrast to acclimatization – that change a plant's phenotype and physiology and make the organism more fit for a specific environment (Novoplansky, 2002). An *adaptive trait* denotes a trait that confers a fitness advantage (Schmitt et al., 1999) and has evolved through natural selection over several generations.
Canopy	The canopy is the aboveground portion of a plant community, formed by the collection of individual plant crowns (Campbell & Norman, 1989). In general, traits describing canopy architecture include the number, size, shape, distribution and orientation of their leaves (Duursma et al., 2012; Niinemets, 2010; Rahman, Duursma, Muktadir, Roberts, & Atwell, 2018) LA = leaf area SLA (specific leaf area) = leaf area / leaf dry weight Leaf inclination angle or petiole angle Light interception = amount of light captured
Competition	Competition describes the negative effects on growth of resource restrictions due to neighbouring organisms (Aphalo, Ballaré, & Scopel, 1999). Intra‐specific competition refers to competition between individuals of the same species, for example in crop monocultures; whereas inter‐specific competition refers to competition between different species, for example crop‐weed competition or naturally mixed‐species vegetations.
Phenotypic plasticity	Phenotypic plasticity is the capacity of an individual plant to express different phenotypes in response to environmental variation (Aphalo et al., 1999; Schmitt et al., 1999; Smith & Whitelam, 1997).
Shade‐avoidance syndrome (SAS)	The shade‐avoidance syndrome refers to the multiple responses of a plant to shade and changes in light quality caused by neighbouring plants (Ballaré & Pierik, 2017; Roig‐Villanova & Martínez‐García, 2016; Smith & Whitelam, 1997): Hyponasty (upward movement of leaf or petiole) Accelerated hypocotyl and internode elongation Increased apical dominance (reduced branching and tillering) Accelerated flowering

First, we will provide a detailed description of light cues in canopies, including how these are perceived and processed by plants to plastically regulate development. This will be based largely on knowledge available from the plant model species *Arabidopsis thaliana*. We then scale up from changes of a single plant to plant communities. We further address the functional consequences of the shade responses and the questions of plasticity and adaptation in this context (Table 1).

## 2 DETECTING LIGHT SPECTRAL CHANGES IN DENSE STANDS

**FIGURE 1:**
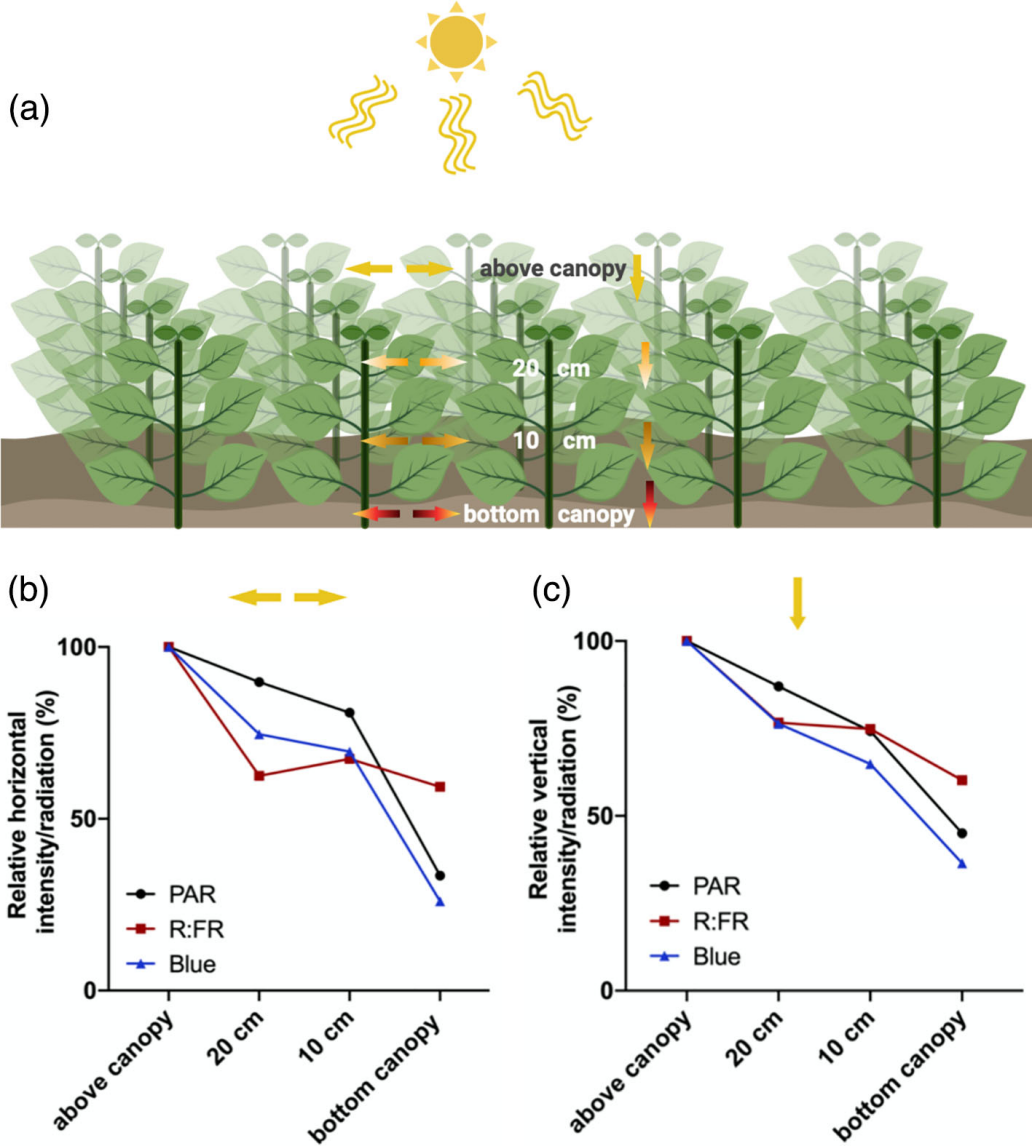
Changes in light quality and quantity in different canopy strata. (a) The cartoon illustrates a basil (*Ocimum basilicum*) canopy in which PAR (photosynthetic active radiation), blue (*λ* = 400–499) and R:FR (red to far‐red ratio; [R (*λ* = 650–670): FR (*λ* = 720–740)]) were measured at different canopy heights. Arrows illustrate the directions of the light measurements at the different heights. (b, c) Quantifications of horizontally (b) or vertically (c) measured PAR (black line), blue (blue line) and R:FR (red line) light at the different canopy heights (above canopy, 20 cm, 10 cm and bottom canopy), expressed as percentage of the values measured above the canopy. The basil canopy consisted of 20 plants that were transplanted 6 days after germination, in a checkerboard pattern with 15 cm distance from each other. The canopy height was 30 cm from soil level. Graphs show light measurements made with a LI‐COR LI‐180 spectrometer, using a cosine corrected sensor, in a 37‐day‐old canopy (*n* = 3). The experiment was performed in the greenhouse facilities of Utrecht University. Created with BioRender.com [Colour figure can be viewed at wileyonlinelibrary.com]

**FIGURE 2:**
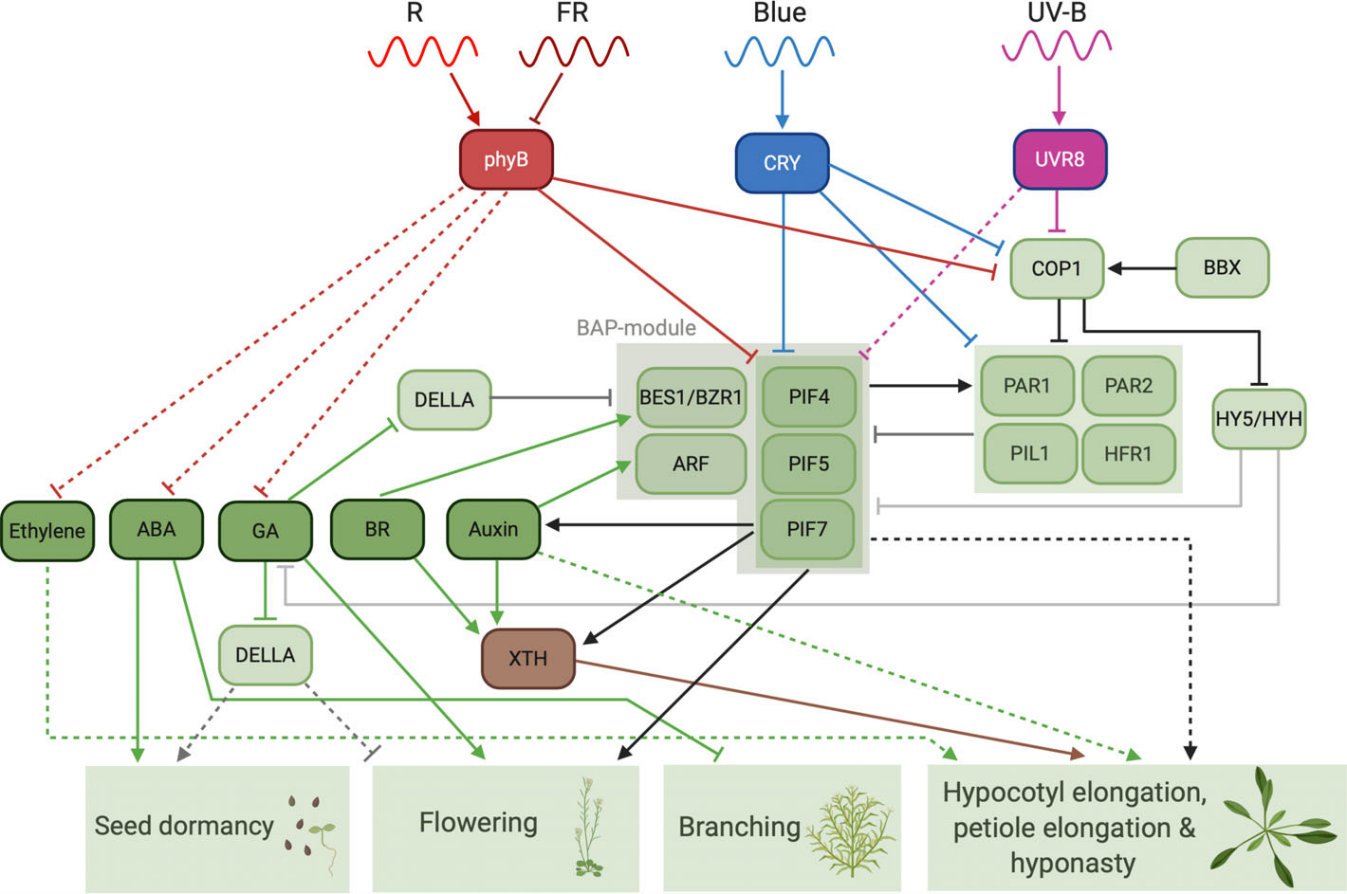
Simplified schematic overview of the signal transduction pathway of shade‐induced seed dormancy, early flowering, reduced branching, hyponasty and accelerated hypocotyl and petiole elongation. Interactions between important proteins (light green) and hormones (dark green) are shown and result in the different SAS responses. See main text for details. Arrows, positive effect; inhibitors, negative effect; solid lines, confirmed interactions/known signalling pathways; dotted lines, exact mechanisms to be elucidated. Created with BioRender.com [Colour figure can be viewed at wileyonlinelibrary.com]

Plants have specific spectral absorption and reflection properties, strongly determined by the absorption properties of chlorophyll. When growing in close vicinity, they therefore collectively alter the light composition inside the vegetation. The earliest light cue reflected from neighbouring plants is a change in the red (R) to far‐red (FR) ratio (R:FR) (Ballaré et al., 1990). Plants absorb blue (400–500 nm) and red (635–700 nm) wavelengths through chlorophyll to fuel photosynthesis while reflecting FR wavelengths (700–780 nm). Sunlight has a R:FR of approximately 1.2, but neighbouring plants can reduce this ratio to as low as 0.1 in deep canopy shade (i.e., canopy closure). Interestingly, R:FR can drop already before true shading occurs (Ballaré et al., 1990), due to reflection of FR light by neighbouring plants that are not yet overlapping (Figure 1). The initial drop in R:FR is, therefore, an early warning cue of upcoming competition for light and is followed by a decrease of total light intensity and depletion of blue light when the canopy further develops and true shading occurs (reviewed in Pierik & Wit, 2014). In the next paragraphs, the changed light quality due to the (closing) canopy will be discussed (summarized in Figure 2). In addition to red, FR, blue and total light intensity, also other factors weigh in, such as the change in light spectrum during the day, the weather, sun flecks and sun zenith angle (Kotilainen et al., 2020). Since these light changes are not caused by neighbouring plants, we do not discuss them in depth in this review and direct the reader to an excellent recent update on the matter by Kotilainen et al. (2020). UV‐B light is a potent antagonist of plant responses to FR light enrichment and blue light depletion and will also be decreased inside vegetation due to absorption. When a shade‐avoiding plant perceives UV‐B through the UVR8 photoreceptor, shade avoidance is inhibited through UVR8 interaction with COP1, resulting in HY5 accumulation (Favory et al., 2009) and through inhibition of PIFs (Hayes, Velanis, Jenkins, & Franklin, 2014; Mazza & Ballaré, 2015). Most mechanistic knowledge discussed here comes from studies on the model species *Arabidopsis thaliana* (Arabidopsis), although some aspects have also been investigated in other species.

### 2.1 Decreased red to far‐red ratio

Plants perceive R and FR light with the phytochrome class of photoreceptors. The dicot species Arabidopsis has five phytochrome genes (*PHYA‐E*), while monocots typically have three (*PHYA‐C*) (Mathews & Sharrock, 1996; Sharrock & Clack, 2002). The phyB receptor is the dominant player in the shade‐avoidance response. Phytochromes are photoreversible proteins that are activated by R light (converting phyB into the active Pfr form), rapidly inactivated by FR light and gradually inactivated in the dark (to the inactive Pr form) (Hillman, 1967; Wang & Wang, 2015). Phytochromes are also sensitive to temperature, and elevated temperature‐mediated conversion of Pfr to Pr is one of the mechanisms through which plants sense temperature (Legris, Ince, & Fankhauser, 2019). PhyA can also stay active in FR light and constitutes a negative feedback that can attenuate shade‐avoidance responses (Yang et al., 2018).

### 2.2 Depletion of total light intensity and blue light

The reduction in R:FR starts when growing plants in a community are approaching each other. However, when the foliage of plants starts overlapping, this in addition entails a decrease in blue light (low blue light), following from light absorption by the overlaying leaves. The overall quantity of light, the photosynthetically active radiation, also decreases inside vegetation during the growth season. This decrease is registered primarily by the cryptochrome blue light receptors but also through the reduced rates of photosynthesis in low light (Millenaar et al., 2009; Mullen, Weinig, & Hangarter, 2006). The photoreceptors sensitive to blue light are, besides the cryptochromes, phototropins and members of the ZEITLUPE family (Pudasaini & Zoltowski, 2013). Phototropins regulate, amongst others, the phototropic response of seedlings and adult plants towards light‐enriched spaces (Briggs & Christie, 2002), whereas the cryptochromes (CRY1 and CRY2) play an active role in elongation responses (Keller et al., 2011; Keuskamp et al., 2011; Pedmale et al., 2016). It is important to note that investigating how plants perceive and respond to the absence of blue light helps to understand the molecular mechanisms of this pathway, but the depletion of blue light alone in a white light spectrum is not a naturally occurring situation. Low blue light in natural conditions will always be accompanied by a decrease in R:FR when indicating canopy shade. Indeed, low R:FR and low blue light pathways converge and are integrated, leading to strong shade avoidance (Wit, Keuskamp, et al., 2016) and regulation of phototropic bending in light‐grown plants (Goyal et al., 2016). In addition to convergent action with phyB‐sensed R:FR cues, cryptochromes themselves also show sensitivity to another wavelength that is abundant inside canopies, green light, in regulating hypocotyl elongation in Arabidopsis (Sellaro et al., 2010).

## 3 PROCESSING CANOPY LIGHT CUES

The perception of canopy‐associated light cues initiates a signalling cascade that differs between early neighbour detection and canopy shade (Hersch et al., 2014). The combination of different shade cues can either lead to signal intensification and enhance a certain pathway or trigger distinct pathways (Wit, Keuskamp, et al., 2016). In the next paragraphs, we will briefly mention the most important components of shade signalling (summarized in Figure 2), resulting in elongation responses of hypocotyls, stems and leaves.

### 3.1 Phytochrome interacting factors

A class of basic helix–loop–helix (bHLH) transcription factors called the *PHYTOCHROME INTERACTING FACTORS* (*PIFs*) interact with both phytochromes and cryptochromes (Leivar & Quail, 2011). Binding of the activated phytochromes or cryptochromes to PIFs inactivates them and in many instances even leads to PIF degradation (Huang et al., 2018; Pedmale et al., 2016). The Arabidopsis genome contains eight different *PIF* genes (*PIF1‐8*), of which *PIF4*, *PIF5* and *PIF7* play a major role in the shade‐avoidance responses (Hornitschek, Lorrain, Zoete, Michielin, & Fankhauser, 2009). Upon binding of active phytochrome, PIFs are phosphorylated and PIF4 and PIF5 are subsequently degraded (Lorrain, Allen, Duek, Whitelam, & Fankhauser, 2008). Although PIF7 is not rapidly degraded, it still gets inactivated upon phosphorylation (Li et al., 2012). Although a role for PIF8 in shade avoidance has not been tested yet, it was recently shown to repress phyA‐dependent light responses (Oh, Park, Song, Bae, & Choi, 2020). PIFs can bind directly to DNA, thereby activating SAS‐related genes such as genes encoding cell‐wall‐modifying enzymes, as well as several growth‐promoting hormones, especially auxin (Hornitschek et al., 2012; Li et al., 2012; Pedmale et al., 2016). These molecular processes allow plants to activate the SAS phenotypes, helping them to reposition their photosynthetic organs towards the light.

### 3.2 Hormones

As mentioned above, PIFs interact with hormone pathways that are involved in shade‐avoidance control. The best studied and core regulatory hormone is auxin (Iglesias, Sellaro, Zurbriggen, & Casal, 2018; Yang & Li, 2017). Although we give a brief coverage here, we point the readers to Küpers, Oskam, and Pierik (2020) for a much more detailed overview of auxin control in shade‐avoidance responses. PIFs directly activate transcription of genes encoding YUCCA enzymes involved in auxin biosynthesis but also of genes encoding auxin transport‐associated proteins and proteins relevant in auxin response, such as AUX/IAAs (Wit, Galvão, & Fankhauser, 2016). Auxin is important in growth and development of almost all plant organs. It has been shown that treatment of Arabidopsis plants with additional FR light, thus creating a low R:FR ratio, leads to increased auxin levels in the shoot (Keuskamp, Pollmann, Voesenek, Peeters, & Pierik, 2010; Li et al., 2012; Procko, Crenshaw, Ljung, Noel, & Chory, 2014; Tao et al., 2008). Applying auxin to seedlings or certain organs often mimics the responses associated with SAS (Chapman et al., 2012; Keuskamp et al., 2011; Xu et al., 2018). Auxin synthesis occurs in different parts of the plant, and auxin transport is needed for the shade‐avoidance responses to occur, both in hypocotyl elongation (Keuskamp, Pollmann, et al., 2010) and hyponasty (Michaud, Fiorucci, Xenarios, & Fankhauser, 2017; Pantazopoulou et al., 2017). Plant organs can also show contrasting growth responses due to differential auxin responsiveness such as between the leaf lamina and petiole (Wit, Ljung, & Fankhauser, 2015). In rice seedlings, auxin‐related genes induced by shade are upregulated in the first leaves, even though the coleoptiles are responding with elongation (Liu, Yang, & Li, 2016). It was shown in *Brassica rapa* seedlings that supplemental FR triggers auxin synthesis in the cotyledons, and auxin is subsequently transported to the hypocotyl to promote elongation (Procko et al., 2014). Shade‐induced auxin synthesis is regulated by PIFs, and PIF action in addition can be further promoted by auxin response itself via auxin response factors (ARFs, transcription factors) that increase PIF‐dependent gene expression (Oh et al., 2014). However, further studies are necessary to elucidate if such positive feedback also regulates shade avoidance. Auxin and PIFs both lead to the upregulation of a group of cell‐wall‐modifying enzymes, xyloglucan endotransglucosylase/hydrolases (XTHs) that allow cell‐wall modifications needed for the changes in cell growth needed for shade avoidance (Keuskamp et al., 2011; Sasidharan et al., 2010). Cell‐wall‐modifying proteins are active at low apoplastic pH, and shade exposure is accompanied by acidification of the Arabidopsis petiole apoplast (Sasidharan et al., 2010). This may very well also be auxin‐dependent, probably via SMALL AUXIN UPREGULATED proteins that activate plasma membrane ATPases (Fendrych, Leung, & Friml, 2016).

In addition to auxin, also gibberellin synthesis is promoted in low R:FR. Gibberellin promotes SAS by causing degradation of the growth inhibiting DELLA proteins (Feng et al., 2008). In non‐shaded conditions, DELLA proteins bind PIFs and thereby inhibit their function (de Lucas et al., 2008; Feng et al., 2008). This is suppressed by low R:FR‐mediated, DELLA degradation via gibberellin (Djakovic‐Petrovic, de Wit, Voesenek, & Pierik, 2007). Another important hormone regulator of SAS is brassinosteroid (Hayes et al., 2019; Keuskamp et al., 2011; Kozuka et al., 2010; Wit, Galvão, & Fankhauser, 2016). Despite the clear evidence for brassinosteroid involvement, the precise mechanisms are still unknown. Different from gibberellin and auxin, brassinosteroid levels do not seem to increase in shade, and there are even reports of reduced brassinosteroid levels in shade compared to control light (Bou‐Torrent et al., 2014; Gommers et al., 2018). At least part of the brassinosteroid involvement occurs via its regulation of BES1/BZR1 transcription factors that interact positively with PIFs to promote target gene expression (e.g., Hayes et al., 2019; Oh et al., 2014). A synergistic relationship between auxin and brassinosteroid has also been proven since both are needed to achieve full hypocotyl elongation under low blue light conditions (Keuskamp et al., 2011). The BZR1‐PIF‐ARF, BAP module is inhibited by DELLA proteins (Oh et al., 2014).

Two other hormones can be involved in shade‐avoidance control, but the molecular mechanisms are less well characterized: ethylene and abscisic acid. Ethylene can promote shoot elongation in a species‐ and conditions‐dependent manner (reviewed in Pierik, Tholen, Poorter, Visser, & Voesenek, 2006). Ethylene is volatile, and its emission is promoted by low R:FR. The hormone can even accumulate in the still air inside a canopy and ethylene‐insensitive transgenic tobacco plants had reduced shade‐avoidance properties (Pierik, Cuppens, Voesenek, & Visser, 2004; Pierik, Whitelam, Voesenek, de Kroon, & Visser, 2004). In water‐adapted terrestrial plants, ethylene has been shown to promote submergence‐induced shoot elongation through downregulation of abscisic acid (Benschop et al., 2005; Hoffmann‐Benning & Kende, 1992). However, the role of abscisic acid in shade avoidance so far has been mostly described for its inhibition of branching under low R:FR (Reddy, Holalu, Casal, & Finlayson, 2013). Although abscisic acid can be a strong inhibitor of low R:FR responses, such as accelerated hypocotyl elongation in Arabidopsis (Hayes et al., 2019), it remains to be investigated if this hormone is part of the intrinsic phyB‐regulated elongation pathways.

### 3.3 Other regulators

Besides PIFs and hormones, there are other positive and negative regulators important in the shade‐avoidance response. ELONGATED HYPOCOTYL5 (HY5) and its homolog HYH are photoreceptor‐regulated via CONSTITUTIVELY PHOTOMORPHOGENIC 1 (COP1) E3‐ligase that targets HY5 and HYH (Pacín, Semmoloni, Legris, Finlayson, & Casal, 2016). The HY5 and HYH proteins inhibit hypocotyl and petiole elongation in Arabidopsis (Nozue et al., 2015). *hy5* mutants show constitutively enhanced hypocotyl elongation, while over‐expression of *HY5* leads to inhibited elongation (van Gelderen et al., 2018). COP1 also interacts with double B‐BOX (BBX) zinc finger transcription factors, of which BBX21 and BBX22 are both involved in early‐ and long‐term SAS responses (Crocco, Holm, Yanovsky, & Botto, 2010). BBX25 interacts with HY5 and enhances COP1 function, lifting the inhibition of the hypocotyl elongation (Gangappa et al., 2013). LONG HYPOCOTYL IN FAR‐RED 1 (HFR1), PHYTOCHROME RAPIDLY REGULATED 1 (PAR1) and PAR2 are negative regulators of the shade‐avoidance responses (Buti, Hayes, & Pierik, 2020). Plants over‐expressing these genes show diminished shade‐avoidance responses, whereas reducing the transcript levels of these genes leads to enhanced shade‐avoidance responses (Hornitschek et al., 2009; Li et al., 2014; Roig‐Villanova et al., 2007; Steindler et al., 1999). It was found that HFR1, PAR1 and PAR2 can interact with PIFs, thereby preventing PIFs from binding to target sequences on the DNA. Indeed, when plants in low R:FR are simultaneously exposed to low blue light, this enhances elongation by suppressing low R:FR‐induced HFR1 protein and *HFR1* gene expression (Wit, Keuskamp, et al., 2016). The most recent insights into this complex network is that another non–DNA‐binding HLH protein, KIDARI (KDR)/PACLOBUTRAZOL‐RESISTANCE6 (PRE6) can physically interact with PAR1 and PAR2, as well as several other growth‐inhibitory proteins, thereby preventing KDR's targets from inhibiting PIF activity (Buti et al., 2020; Buti, Hayes, & Pierik, 2020). Molecular regulators in species other than Arabidopsis have been thoroughly discussed in reviews by Kebrom and Brutnell (2007) and Carriedo, Maloof, and Brady (2016).

## 4 DEVELOPMENTAL PLASTICITY IN RESPONSE TO LIGHT CUES

Shade‐avoidance responses help plants to grow away from shaded zones in the canopy, into the more light‐exposed areas, enabling photosynthesis and consequently growth. Since resources are limiting in dense communities, growth trade‐offs between different organs become inevitable. In this section, we will discuss different plant traits underlying SAS that are also summarized in Figure 2.

### 4.1 Hypocotyl, petiole and stem elongation

In early canopies, seedlings can already detect neighbours and change their growth forms accordingly. This mostly shows by enhanced elongation of the hypocotyl and reduced growth of the cotyledons. Exposure to the combination of supplemental FR and blue light depletion (low blue) causes an enhanced hypocotyl elongation compared to their separate treatments (Wit, Keuskamp, et al., 2016). In adult Arabidopsis rosette plants, supplemental FR causes petiole and stem elongation (Gommers et al., 2017; Sasidharan et al., 2010). Besides Arabidopsis, supplemental FR elicits internode and stem elongation in stem‐forming plants, such as tobacco, sunflower, soybean, spring wheat, maize, tomato, alfalfa and *Powell amaranth* (Brainard, Bellinder, & DiTommaso, 2005; Caton, Cope, & Mortimer, 2003; Chitwood et al., 2015; Evers, Andrieu, & Struik, 2006; Green‐Tracewicz, Page, & Swanton, 2011; Lorenzo et al., 2019; Page, Tollenaar, Lee, Lukens, & Swanton, 2009; Wille, Pipper, Rosenqvist, Andersen, & Weiner, 2017). Exposure to low blue light alone does not necessarily cause a change in petiole elongation in Arabidopsis compared to white light, suggesting that petiole elongation is regulated via different pathways or in a different manner in this species as compared to low R:FR‐driven elongation (Pierik, Djakovic‐Petrovic, Keuskamp, de Wit, & Voesenek, 2009), but see Keller et al. (2011). Low blue light alone does stimulate internode elongation in various other species, including *Stellaria longipes* (Sasidharan, Chinnappa, Voesenek, & Pierik, 2008), tobacco (Pierik, Whitelam, et al., 2004), *Sinapis alba L*. and *Datura ferox L*., and the strongest elongation in *D*. *ferox L*. occurred under combined low R:FR and low blue light levels (Ballaré, Scopel, & Sanchez, 1991).

### 4.2 Hyponasty

Another phenotypic characteristic of shade avoidance is the upward movement of leaves (hyponasty) that typically occurs in rosette plants, such as Arabidopsis. Shade‐induced hyponasty leads to a higher leaf lamina position in a canopy, thus preventing chances of being shaded by neighbouring leaves. Hyponasty is typically induced by low R:FR and exposure of just the leaf tip to supplemental FR is already enough to initiate hyponasty through auxin synthesis in the leaf tip and subsequent transport to the petiole (Michaud et al., 2017; Pantazopoulou et al., 2017). Nevertheless, other light cues such as low photosynthetic active radiation can also induce strong upward leaf movement in an auxin‐dependent manner (Millenaar et al., 2009). Interestingly, prior to the plant sensing the changed light situation with its photoreceptors, the physical touching of adjacent leaf tips can also trigger hyponasty in dense Arabidopsis monocultures (Wit et al., 2012). The touch‐induced hyponasty does not seem to occur though the canonical low R:FR‐dependent regulators, but the molecular mechanisms underpinning this response are still to be resolved.

### 4.3 Apical dominance

Plants that have multiple shoot branches, or tillers in grasses, show inhibition of branching or tillering under shaded conditions (Casal, Sanchez, & Deregibus, 1986; Caton et al., 2003; Green‐Tracewicz et al., 2011; Wang et al., 2013). The *Tb1* gene in maize and orthologs in other species regulates this apical dominance (Doebley, Stec, & Gustus, 1995; Takeda et al., 2003). Mutants in these genes cause plants to tiller in both control and shaded conditions (Kebrom, Burson, & Finlayson, 2006). *PhyB* mutation in sorghum causes reduced tillering as well, showing a direct link between light perception and changes in tillering (Kebrom et al., 2006).

### 4.4 Life cycle adjustments

Although many species display strong phenotypic plasticity to shade cues, others may not necessarily change their architecture but avoid competition through life cycle tactics such as early flowering or delayed germination. Shade can prolong seed dormancy to ensure germination in favourable light conditions (Casal, Sanchez, Benedetto, & Miguel, 1991; Cumming, 1963; Poppe & Schäfer, 1997; Vazquez‐Yanes & Smith, 1982). Exposing seeds to supplemental FR light for example prevents germination while treating seeds with a period of red light lifts the dormancy (Debeaujon & Koornneef, 2000; Lee & Lopez‐Molina, 2012; Piskurewicz et al., 2008). Germination of these species with light‐sensitive germination depends on a stable pool of phyB, and to achieve this, a period of R light is required. Downstream of phyB, ABA and GA controls seed dormancy and seed germination, respectively (Devlin et al., 1999; Lee et al., 2012; Lee & Lopez‐Molina, 2012; Piskurewicz et al., 2008). Shade also causes changes in the later life stages of plants. For annual plants such as Arabidopsis, early flowering is an established SAS trait (Cerdán & Chory, 2003). This early flowering is regulated through *PIF4*, *PIF5* and *PIF7* downstream of PhyB (Galvāo et al., 2019). It is also at least in part regulated through GA, since silencing GA biosynthesis genes causes late flowering in both control and supplemental FR conditions (Hisamatsu & King, 2008). Interestingly, the perennial species alfalfa (*Medicago sativa*) exhibits delayed flowering upon low R:FR treatment, indicating an uncoupling of the shade‐avoidance responses and early flowering (Lorenzo et al., 2019), tentatively associated with different life cycle durations.

## 5 SHADE AVOIDANCE FROM A PLANT COMMUNITY PERSPECTIVE

**FIGURE 3:**
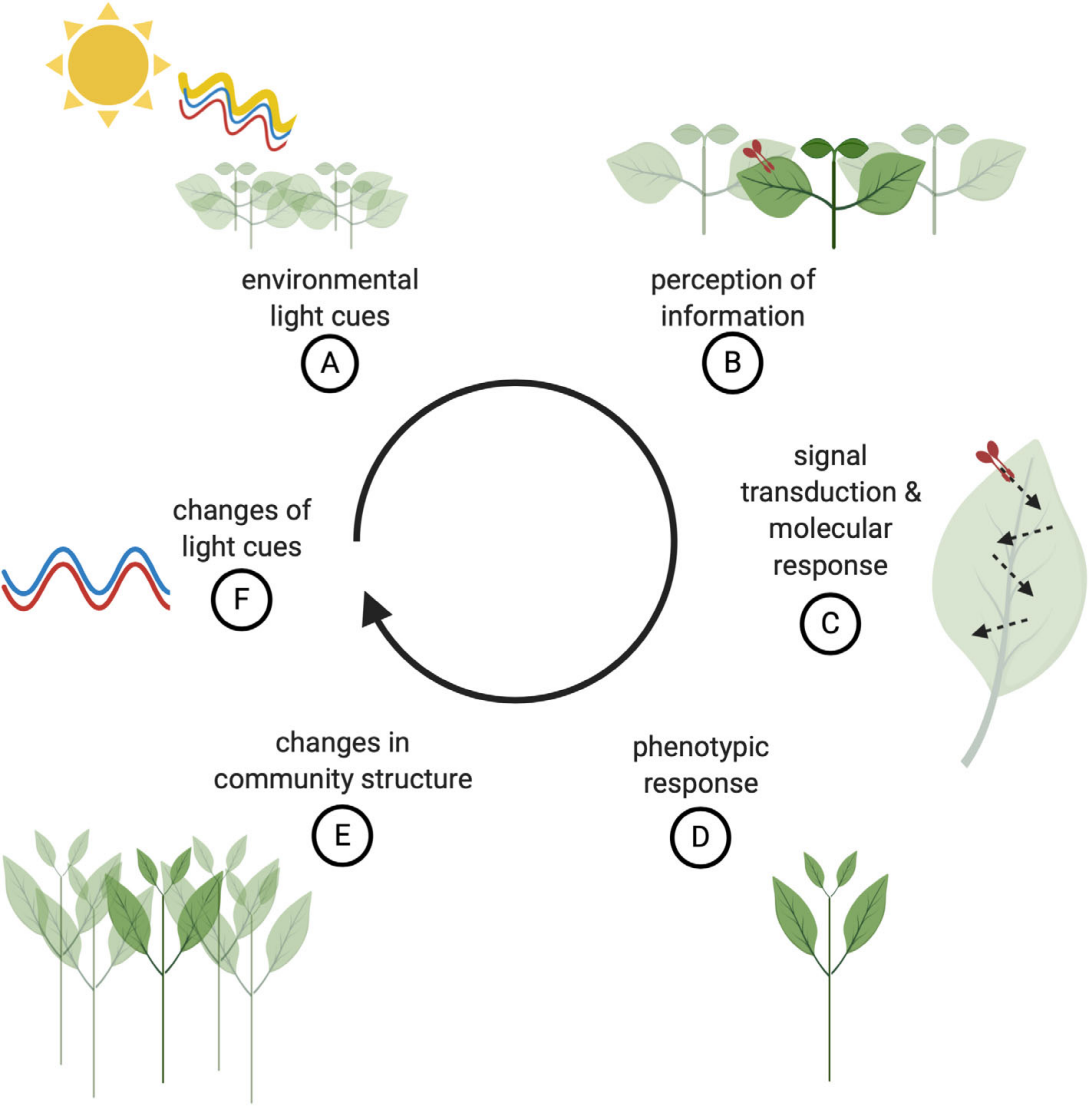
Dynamic between light cues and plant architecture. (A) Environmental light cues are (B) perceived by the plant. (C) Signal transduction pathways evoke specific molecular changes and lead to (D) phenotypic responses. (E) This entails architectural changes in the canopy of a plant community, (F) in turn changing the light quality and quantity in a canopy. This loops back in an ongoing feedback of changes in light cues and architecture. Created with BioRender.com [Colour figure can be viewed at wileyonlinelibrary.com]

Vegetation is formed by multiple individuals, often from different species, with different sets of response abilities that together shape the 3D structure of a canopy. Here, we will integrate the mechanistic knowledge of SAS from the individual plant to the plant community level (Figure 3). We will mostly focus on homogeneous annual plant canopies but will also briefly discuss more complex canopies of mixed species and height stratification. The canopy architecture of a plant community is very dynamic, since it is built by different individuals that may display different plasticities to neighbour proximity. As a consequence, the canopy architecture is highly dynamic.

### 5.1 How does SAS affect canopy architecture?

SAS responses are triggered by shade cues described earlier and lead to modifications of the canopy architecture. Once the canopy architecture changes, this in turn affects the light quality distribution throughout the canopy, thus changing the very light cues that set‐in motion the density‐induced changes in the canopy architecture, in turn adjusting the individual responses (Figure 3).

Shade‐avoidance responses are a way for plants to forage for light and avoid shaded patches. Phototropism directs plant organs through bending towards light patches in the canopy (Ballaré, 1999; Ballaré, Scopel, Roush, & Radosevich, 1995; López Pereira, Sadras, Batista, Casal, & Hall, 2017). Vertical growth is promoted via hypocotyl, petiole, internode and stem elongation and allows access to higher canopy layers (Schmitt, 1997; Schmitt et al., 1999). To further increase light interception, plants optimize leaf positioning, form new leaves on the upper part of the plant and senesce old leaves on the lower parts (Boonman et al., 2006; Boonman, Prinsen, Voesenek, & Pons, 2009; Maddonni, Otegui, Andrieu, Chelle, & Casal, 2002; Pantazopoulou et al., 2017; Pantazopoulou, Bongers, & Pierik, 2020). A more open canopy structure is generated by consequences of apical dominance with reduced branching and less tiller and leaf formation. Furthermore, leaf angle adjustments from relatively horizontal to a more vertical orientation in response to shade cues further opens the canopy allowing more light penetration down to the lowest regions (Pantazopoulou et al., 2020). The enhanced shoot elongation and senescence come with a trade‐off of less photosynthetic active tissue and a lower leaf to stem biomass ratio. In addition, investment in petiole elongation often leads to a reduction in leaf area (Bongers et al., 2019; Wit et al., 2015). A more open canopy facilitates light penetration deeper into the canopy, allowing better photosynthesis in the lower leaves. This can stimulate whole‐plant photosynthesis, but it can of course also foster growth of competing plants at the bottom of the canopy (Box 1).

Box 1 **Agricultural implications**

Generally, SAS responses are viewed as undesirable traits in agriculture for their negative effects on yield. This is mainly due to the changes in biomass allocation (Carriedo et al., 2016; Kebrom & Brutnell, 2007; Liu et al., 2016; Roig‐Villanova & Martínez‐García, 2016; Wille et al., 2017; Wit et al., 2018). Redirecting resources to shade‐responsive tissues such as internodes and stems go at the expense of roots, flowers, fruits and seeds. The investment in non‐harvestable organs leads to a decrease in crop yield (Boccalandro et al., 2003; Morgan, Finlayson, Childs, Mullet, & Rooney, 2002; Page, Tollenaar, Lee, Lukens, & Swanton, 2010; Robson, McCormac, Irvine, & Smith, 1996). This is the case for some of the most economically important crops such as cereals (Garg et al., 2006; Page et al., 2009) and many vegetables such as tomato or soybean (Wu et al., 2017).

Other negative impacts of SAS in agriculture are increased lodging (Schmitt, McCormac, & Smith, 1995), reduced tuberization, for example, in potatoes (Boccalandro et al., 2003; Jackson & Prat, 2008) and early flowering in crops from the *Brassicaceae* family, such as cabbage and kale and *Asteraceae* family like lettuce (Meng, Kelly, & Runkle, 2019). Therefore, suppressing the SAS‐induced elongation responses seems an obvious solution in crop monocultures to enhance the harvest index, since more resources would be allocated to harvestable organs (Liu et al., 2016; Robson et al., 1996; Smith, 1995; Wit et al., 2018; Yang, Seaton, Krahmer, & Halliday, 2016).

Furthermore, the previously mentioned SAS responses create a more open canopy structure, that is, a canopy structure that allows more light to penetrate to the leaves in the lowest regions, thereby also facilitating weed growth in the lower zones. One way to counteract this would be to increase sowing density and sowing uniformity (Lu et al., 2020; Weiner et al., 2010) which will lead to a more rapidly closing canopy and stronger light extinction. Alternatively, planting of weed‐competitive phenotypes that, for example inhibits weeds from growing and even preventing them from germinating, would also inhibit weed proliferation (Andrew, Storkey, & Sparkes, 2015; Brainard et al., 2005; Mahajan & Chauhan, 2013; Pickett et al., 2014; Raj & Syriac, 2017; Seavers & Wright, 1999; Worthington & Reberg‐Horton, 2013). Such weed‐competitive phenotypes might consist of horizontal leaves that cast intense shade and high levels of branching/tillering: the opposite of shade avoidance (Pantazopoulou et al., 2020).

Suppression of hyponastic leaf movement might effectively reduce light penetration inside the canopy and at the same time maximize the leaf surface of canopy plants for better photosynthesis, that is, increase their biomass. Indeed, a recent study confirmed that dense stands of non‐hyponastic *pif7* Arabidopsis mutants had improved rates of canopy closure and suppression of invading competitors as compared to wildtype stands at the same density (Pantazopoulou et al., 2020). Crop orthologs of the Arabidopsis *PIF7* gene may thus constitute interesting targets for leaf angle manipulations in crops to improve growth and weed suppression.

Modifications in the structure or the number of tillers in cereals would be another way to enhance canopy closure. Upon shade, inactivation of phyB in cereals leads to accumulation of TEOSINTE BRANCHED 1 (TB1) which in turn activates GRASSY TILLERS 1 (GT1), a class I HD‐ZIP transcriptional regulator that suppresses tillering (Carriedo et al., 2016; Kebrom, Brutnell, & Finlayson, 2010; Whipple et al., 2011). Tillering control under high‐density shade cues would be another interesting target for cereal breeding towards weeds suppression and crop yield optimization.

It is important to mention that severely suppressing SAS could entail undesired side effects. First, completely inhibiting SAS would also mean impeding the capacity for balancing size inequalities (Pantazopoulou et al., 2020; Weiner et al., 2010; Weiner & Freckleton, 2010) such as *phyb* mutant in wheat which showed severely reduced stem elongation (Pearce, Kippes, Chen, Debernardi, & Dubcovsky, 2016). Second, whether the elongation response goes at the expense of yield or not depends on what the harvested organ of the crop is. For example, in biofuel crops, such as *Miscanthus giganteus*, where an increase in shoot biomass is key, it is less relevant which organs have relatively increased or decreased (Danalatos, Archontoulis, & Mitsios, 2007; Warnasooriya & Brutnell, 2014). Each crop has a different canopy architecture, so SAS reduction has to be in agreement and respect of SAS‐phenotypic characteristics of each crop plant, in order to increase crop yield and potentially suppress weeds more effectively.

Thus, plant responses to density change the canopy architecture, allowing for better light penetration and escape from shaded patches. Along with these changes, the canopy light cues are highly dynamic too (Figure 1). The higher in a canopy, the less red and blue light have been absorbed by neighbouring plants, resulting in a higher R:FR and higher fluence rate of blue and total photosynthetic active radiation. If a plant reaches the top of the canopy, leaves will receive nearly full sunlight but still perceive some FR enrichment from horizontal reflection by proximate neighbours that have grown to similar height. This means that tissues from the same plant are experiencing different light cues at different strata of the canopy. Based on the integration of this information, a plant can fine‐tune its responses (Ballaré & Pierik, 2017; Küpers, van Gelderen, & Pierik, 2018).

### 5.2 Shade avoidance and size‐asymmetric competition at high planting density

**FIGURE 4:**
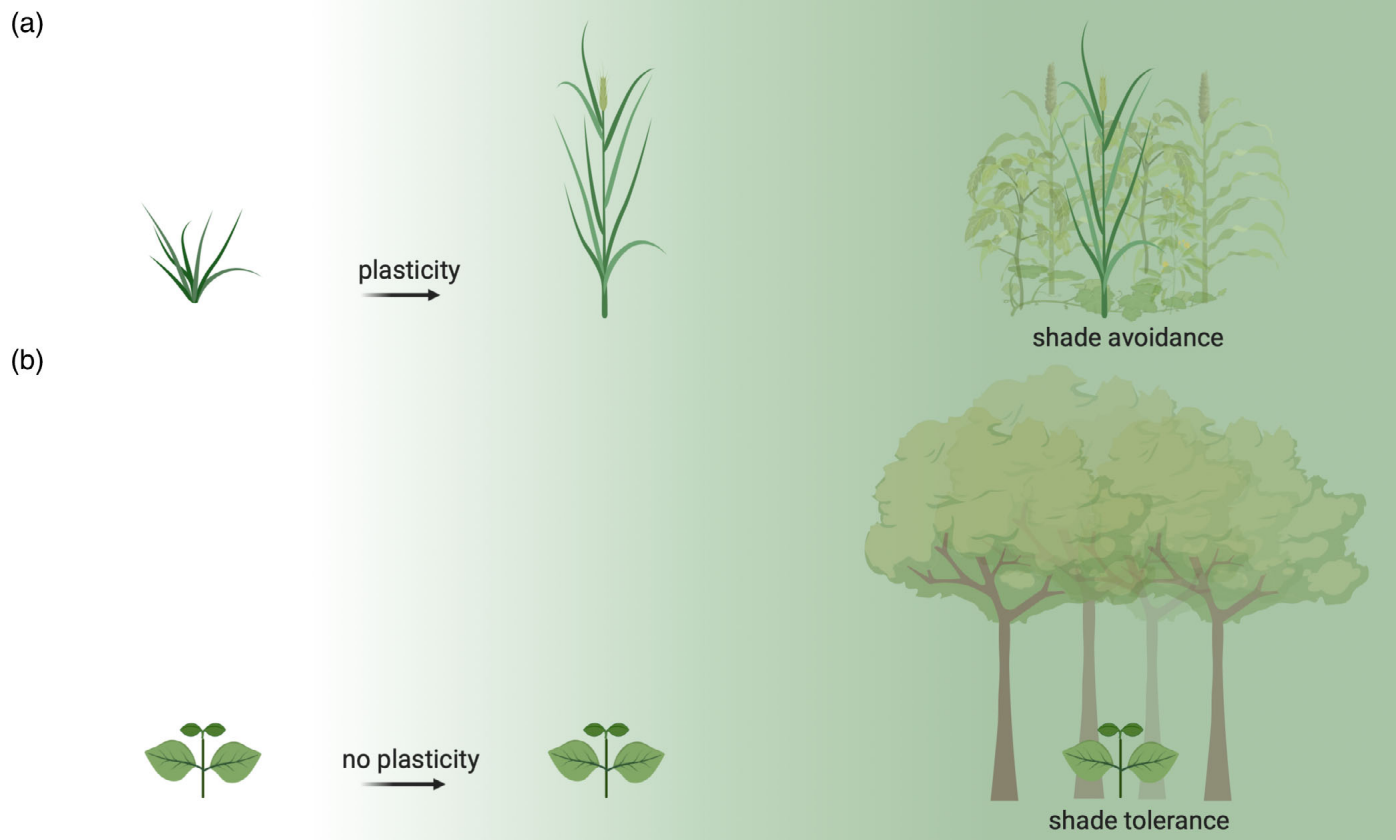
Phenotypic plasticity for density‐associated shade cues. (a) Plants able to respond to increasing neighbour density show plastic SAS responses in contrast to (b) plants that are not responding to density signals and therefore not showing plastic changes in their phenotype. SAS responses are adaptive in competitive fields with approximately equally sized individuals (a). Shade avoidance is typically suppressed in forest understory plants that cannot outgrow the much larger trees around them (b) and here shade tolerance then becomes the adaptive trait. Created with BioRender.com [Colour figure can be viewed at wileyonlinelibrary.com]

In a plant community, size inequalities will always occur, and smaller plants suffer relatively more shading than larger individuals who may be able to reach direct light. Since light typically comes from above, competition for light is size‐asymmetric: a slightly taller individual will absorb a larger fraction of the incoming light as compared to a slightly shorter individual (Weiner, 1985; Weiner & Freckleton, 2010). Thus, the benefits of height growth are disproportional to the height difference between individuals. Shade‐avoidance responses, however, tend to work against the development of size inequalities since the shorter individuals that are in the lower canopy layers experience the strongest shade cues (Figure 1) (Aphalo et al., 1999; Ballaré, 1999; Ballaré & Scopel, 1997; Ballaré, Scopel, & Sánchez, 1997) (Figure 4). Stronger cues tend to elicit stronger responses (Wit, Keuskamp, et al., 2016) and thus especially the suppressed plants show the most pronounced elongation responses, therefore improving their position for light capture. This was illustrated in an elegant study on tobacco plants over‐expressing *PHYA* that show a reduced morphological responsivity to supplemental FR light or neighbours (Ballaré, Scopel, Jordan, & Vierstra, 1994) because phyA represses SAS responses (Ballaré et al., 1994). Size inequalities in a neighbour‐insensitive *PHYA*‐overexpressing monoculture increased much more steeply with density than in wildtype monocultures at the same densities, indicating that SAS helps especially the suppressed plants to improve their competitive position (Ballaré et al., 1994). The SAS morphology improves individual plant performance under high density since it facilitates escape from the shade cast by neighbouring plants. This is advantageous for an individual plant, enhancing its Darwinian fitness (Schmitt, Stinchcombe, Heschel, & Huber, 2003; Weiner, 2019; Weiner, Andersen, Wille, Griepentrog, & Olsen, 2010; Weiner & Freckleton, 2010) (Figure 4).

If the plant community is composed of only one species, either naturally or by human determination, what advantages does it give to display SAS responses if all neighbouring plants do the same? Resources would then be invested in shade avoidance, but they do not return a benefit to the individual plants since their neighbours achieved the very same (Weiner, 2019; Weiner et al., 2010; Weiner & Freckleton, 2010). At the community level, this is a waste of resources into non‐harvestable or non‐productive organs (Weiner et al., 2010) (Box 1). At the same time, not all individuals will be entirely identical in height, and the slightly shorter individuals will be suppressed disproportionately because of the size‐asymmetric nature of competition for light. When densities are very high, density‐induced mortality of suppressed seedlings can occur. Under such conditions, the resources available are used less efficiently for the plant community as a whole, since some of the acquired resources are lost again upon mortality (Lu, Jiang, & Weiner, 2020; Weiner et al., 2010). Since size‐inequalities and mortality are partly counteracted by SAS responses, the community productivity might still benefit from the expression of some degree of SAS responses by the suppressed individuals (Aphalo et al., 1999).

## 6 THE ADAPTIVE VALUE OF SHADE AVOIDANCE

Since environmental conditions are constantly changing, it is essential for plant survival to be able to respond to these changes through phenotypic plasticity (Schmitt, 1997) (Table 1), and shade avoidance is a classic example of this. But why would SAS have to be plastic (Figure 4), would it not be better to always grow maximally tall? The morphological changes involved in shade avoidance enhance a plant's performance at high density when it is growing in a field together with other plants. It can then escape from the shade created by other plants, ensuring photosynthesis. However, when densities are lower and competition for light is weak or even absent, a shade‐avoidance phenotype would confer a fitness disadvantage: a constitutively shade‐avoiding plant would be thin and elongated, thereby lodging easily. It would also not form the branches it needs at low densities to grow vigorously (Ballaré et al., 1995; Schmitt, Mccormac & Smith, 1995). This argument also explains why SAS as a plastic response is important in open fields (Bongers et al., 2019), where competition intensity varies strongly with seasons; early in the year, there is hardly any competition, and SAS would be disadvantageous, whereas later on a plant will experience increasing numbers and sizes of competitors and expressing SAS becomes advantageous. In addition, since SAS comes with trade‐offs, such as reduced lamina size, plasticity allows the investments to be made when necessary, but costs prevented when not needed (Figure 4a). In the earlier sections, we focussed mostly on canopies of relatively similarly sized individuals and mostly took plant density as the dominant variable. In the following sections, we will discuss how SAS expression may vary between different types of canopies and relative plant positions therein, as well as with different additional variables affecting plants, in order to be adaptive.

### 6.1 Why is SAS adaptive?

In order for a plastic response to be adaptive, the phenotype displayed in a certain environment must lead to a fitness advantage in that environment relative to alternative phenotypes (Schmitt, 1997), thus SAS must also result in a fitness advantage to be an adaptive trait (Figure 4). This has indeed been confirmed in various studies (Dudley & Schmitt, 1996; Schmitt et al., 1999; Schmitt, Mccormac, & Smith, 1995), and it was shown that too strong or too weak phenotypic changes in response to neighbour presence reduce fitness in dense stands (Dudley & Schmitt, 1995; Keuskamp, Sasidharan, & Pierik, 2010; Pierik et al., 2003; Weijschedé, Martínková, De Kroon, & Huber, 2006; Weinig, 2000a). Naturally, vegetations differ in the intensity of competition for light, and so do species in their ability to respond to this. For example, variation in low R:FR‐induced stem elongation rate has been documented between (Gilbert, Jarvis, & Smith, 2001; Gommers et al., 2017; Molina‐Contreras et al., 2019; Morgan & Smith, 1978) and even within (Filiault & Maloof, 2012; Sasidharan et al., 2008) species. These observations suggest that natural selection can favour specific degrees of shade‐avoidance potential in different habitats. The ability to respond to shade in a plastic way, and to modulate the intensity of this response depending on the precise environmental conditions, allows plants to grow in a relatively wide range of habitats. Indeed, it has been proposed that plasticity acts against the evolution of ecological specialists (Weinig, 2000a). Studies with mutants impaired in R:FR perception show that they are less efficient than the corresponding wild types at foraging for light in heterogeneous light environments, providing direct evidence for the adaptive value of phytochrome‐mediated shade avoidance (Ballaré et al., 1995; Schmitt et al., 1995, 1999). It has also been shown that an elongated phenotype due to SAS responses increases fitness over non‐elongated plants when growing at high density but reduces fitness at low densities (Aphalo et al., 1999; Donohue, Messiqua, Pyle, Shane, & Schmitt, 2000; Dudley & Schmitt, 1996). In conclusion, although in a dense vegetation, it is the exact position in the canopy that determines a plant's light interception and thus fitness, the ability to be plastic for traits that determine leaf positioning in a canopy allows for optimal light foraging and promotes fitness (Bongers et al., 2019).

### 6.2 Is SAS always adaptive?

Although the fitness advantage of SAS is clear, this does probably depend on such factors as size relative to neighbouring plants, phylogenetic background, ontogenetic stage and the current physiological state. In addition, many other aspects of the environment determine the range of response: population density, availability of resources other than light, time of the year and the type of habitat. For example, a pioneer vegetation of rapidly cycling species that is establishing on bare soil may rely less on phenotypic plasticity, then later successional stages (Lundgren & Sultan, 2005; Weijschedé et al., 2006; Weinig, 2000a, 2000b).

There are also naturally occurring scenarios, where shade avoidance is non‐adaptive for specific species. Under some of these conditions, specialization is favoured over plasticity (Weinig, 2000a). In strongly light‐deprived habitats, for example, in a forest understory as an herbaceous plant or even for small weeds in a crop field (Weinig, 2000b), the situation is different than in an open grassland (Figure 4b). Here, following the SAS escape strategy is unlikely to improve light interception, since outgrowing the neighbouring plants is impossible. Plants adapted to such a forest understory habitat evolved mechanisms to suppress SAS responses and developed ways to be shade‐tolerant (Gommers, Visser, St Onge, Voesenek, & Pierik, 2013; Valladares & Niinemets, 2008). Although shade‐tolerant plants are typically considered to have very low plasticity (Valladares & Niinemets, 2008), they do show some shade responses, such as an increased specific leaf area and a decreased chlorophyll a/b ratio to optimize light harvesting with minimal carbon investments (Evans & Poorter, 2001; Gommers et al., 2013). It appears that shade‐tolerant plants can still sense shade cues but have evolved mechanisms to suppress SAS. Although the molecular mechanisms regulating alternative shade responses are largely unknown, a few recent studies have started to unravel the molecular pathways towards shade‐avoidance suppression. In a comparative study on two *Geranium* species with antithetical shade responses, the shade‐tolerant plant *G*. *robertianum* was found to be able to respond to low R:FR, but within a few hours, reverse its response and suppresses elongation growth in low R:FR. A candidate regulator of this response flexibility is the atypical HLH protein KIDARI (KDR) that seems to promote shade avoidance in shade‐intolerant plants (Gommers et al., 2017), by interacting with other HLH proteins that suppress PIF activity (Buti, Hayes, & Pierik, 2020). Another plant that does not elongate its hypocotyls in response to low R:FR is *Cardamine hirsuta*, and this is associated with a hyperactive phyA photoreceptor that typically antagonizes phyB‐mediated shade‐avoidance responses (Molina‐Contreras et al., 2019). A *phyA* (*sis1*) mutant in this species completely restored hypocotyl elongation in response to low R:FR (Molina‐Contreras et al., 2019). These studies indicate that both in *C*. *hirsuta* and in *G*. *robertianum*, the shade‐avoidance machinery is preserved but mechanisms have evolved to suppress it.

Extreme habitats like alpine vegetations, wetlands or saline soils impose strong additional environmental stresses on plant growth, which might overrule the SAS responses (Keuskamp, Sasidharan, & Pierik, 2010). A naturally occurring example of a genotype not expressing SAS in response to low R:FR is the alpine ecotype of *Stellaria longipes* (Sasidharan et al., 2008). This genotype was collected from alpine sites in the Rocky Mountains where vegetation is extremely sparse and no competition for light occurs. In such environments, plants are typically very short and compact to protect them from the extreme weather conditions. It turns out that this ecotype has lost the ability to elongate in response to low R:FR, whereas a prairie ecotype growing nearby in the lower altitude grasslands is highly responsive to this shade cue (Kurepin, Pharis, Neil Emery, Reid, & Chinnappa, 2015; Sasidharan et al., 2008). Consistently, while the prairie ecotype upregulates cell‐wall loosening through expansins in response to low R:FR, this does not occur in the alpine ecotype. Severe low light treatments still elicited internode elongation in both the ecotypes, accompanied by strong induction of several *EXPANSIN* genes (Sasidharan et al., 2008). Since PIF proteins are known to regulate *EXP* gene expression, the observed variations between the two *Stellaria* ecotypes might suggest differences in PIF activity between the ecotypes, but this remains to be investigated.

Next to loss of shade avoidance through evolutionary adaptation, shade‐avoidance responses can also be suppressed by local environmental conditions occurring within the lifetime of an individual. A recent example is on abiotic stress, where it was found that exposure to very mildly elevated salt concentrations in the soil inhibits low R:FR‐induced hypocotyl elongation. This occurs via an ABA‐dependent inhibition of the brassinosteroid‐dependent transcription factor BES1 (Hayes et al., 2019). Tentatively, suppressing shade avoidance is important for abiotic stress tolerance by maintaining a relatively small shoot. It will be interesting to study if this is a common feature of other abiotic stresses interacting with plant–plant signalling. At least one other factor, UV‐B light, has been shown to also suppresses low R:FR response (Hayes et al., 2014; Mazza & Ballaré, 2015) although this mostly indicates intricate light information integration for optimal light foraging, rather than stress interaction with low R:FR response.

## 7 FUTURE PERSPECTIVES

In this review, we explored the tremendous ecological and agricultural importance of SAS and revealed the complex regulation of the molecular pathways associated to it.

An important aspect for future studies is related to light cue heterogeneity at the (sub)organ level, especially for stem‐forming plants receiving different light information from leaves at different heights. It would be important to unravel how this information is integrated at a whole‐plant level, if and how self‐shading can be distinguished from neighbour plants (Pantazopoulou et al., 2017; Zhang et al., 2020), and how local responses are integrated with systemic responses. Therefore, a major current challenge in shade‐avoidance research is to study the molecular mechanisms underpinning the multiple interactions between different light‐responsive pathways, for example, R:FR versus blue, and the multiple spatial scales within a plant that senses different light environments. Finally, these already complicated interactions have a very strong temporal component since the canopy develops, causing strong temporal fluctuations of neighbour cues. Studies are needed to understand and predict the reliability of cues that are heterogeneous in time and space.

Despite these open questions, the existing knowledge of SAS from the model plant Arabidopsis should already be translated to crops (Box 1). Such translational studies could explore if similar mechanisms are valid for other species and how to adjust them. To create an optimally performing crop plant, rather than entirely suppressing SAS via manipulating the photoreceptors, more subtle approaches might be more promising. Some studies suggest that by targeting downstream effectors of photoreceptors, SAS responses could for instance be limited to a certain developmental stage (Carriedo et al., 2016; Roig‐Villanova & Martínez‐García, 2016), only affecting specific architectural traits (Devlin, Yanovsky, & Kay, 2003; Wei, Zhao, Xie, & Wang, 2018). It would be very interesting to match concepts from Evolutionary Agroecology/Darwinian Agriculture (Denison, 2012; Weiner et al., 2010), where inhibition of SAS in crop monocultures is proposed to inhibit weed proliferation through enhanced closure of the crop canopy, with the molecular–genetic knowledge and tools for shade‐avoidance modulation in Arabidopsis. It might then be possible to target specific genetic loci to select cooperative crops with enhanced communal weed suppression properties.

In vegetable horticulture, the detailed knowledge about SAS pathways can be used not only to target the crop but also the greenhouse light conditions, using LED light spectra (e.g., Demotes‐Mainard et al., 2016) to steer architecture and yield.

## CONFLICT OF INTEREST

The authors declare no conflict of interest.
